# Emergence of New Norovirus Strain GII.4 Sydney — United States, 2012

**Published:** 2013-01-25

**Authors:** Leslie Barclay, Mary Wikswo, Nicole Gregoricus, Jan Vinjé, Ben Lopman, Umesh Parashar, Aron Hall, Eyal Leshem

**Affiliations:** Div of Viral Diseases, National Center for Immunization and Respiratory Diseases; EIS officer, CDC

Noroviruses are the leading cause of epidemic gastroenteritis, including foodborne outbreaks, in the United States (1). Hospitalization and mortality associated with norovirus infection occur most frequently among elderly persons, young children, and immunocompromised patients. Noroviruses belong to the family *Caliciviridae* and can be grouped into five genogroups (GI through GV), which are further divided into at least 34 genotypes. Human disease primarily is caused by GI and GII noroviruses, with most outbreaks caused by GII.4 strains (1). During the past decade, new GII.4 strains have emerged every 2–3 years, replacing previously predominant GII.4 strains. Emergence of these new norovirus strains has often, but not always, led to increased outbreak activity. For example, the previously dominant GII.4 New Orleans strain was not associated with increased norovirus outbreak activity in the United States (2). CDC collects information on norovirus strains associated with outbreaks in the United States through an electronic laboratory surveillance network called CaliciNet (3). This report documents the recent emergence of a new GII.4 strain, GII.4 Sydney, which caused most (53%) of the norovirus outbreaks reported through CaliciNet during September–December 2012. Continued surveillance will enable further assessment of the public health implications and significance of this new strain.

In March 2012, a new GII.4 norovirus strain was identified in Australia. Named GII.4 Sydney, this emergent strain has since caused acute gastroenteritis outbreaks in multiple countries (4). In the United Kingdom, an early onset of the 2012 winter norovirus season was reported in association with emergence of GII.4 Sydney as the dominant strain implicated in outbreaks.* In the United States, GII.4 Sydney has spread rapidly nationwide, causing an increasing number of outbreaks. During September–December 2012, a total of 141 (53%) of the 266 norovirus outbreaks reported to CaliciNet were caused by GII.4 Sydney. The other outbreaks were caused by 10 different GI and GII genotypes, including GII.4 New Orleans. A statistically significant increase in the proportion of outbreaks caused by GII.4 Sydney was noted: four (19%) of 21 outbreaks in September 2012; 22 (46%) of 48 in October 2012; 70 (58%) of 120 in November 2012; and 45 (58%) of 77 in December 2012† (chi-square test for trend; p<0.01). Most (72 [51%]) of these GII.4 Sydney outbreaks resulted from direct person-to-person transmission; 29 (20%) were foodborne, one (1%) was waterborne, and the transmission mode was unknown in 39 (28%) of the outbreaks. Long-term–care facilities and restaurants were the most frequently reported settings, accounting for 91 (65%) and 18 (13%) of the GII.4 Sydney outbreaks, respectively. During the three previous winters, the peak in reported norovirus outbreaks occurred in January; therefore, at present, it is too early to make an assessment of the relative magnitude of the current season.

GII.4 noroviruses remain the predominant cause of norovirus outbreaks, and the GII.4 Sydney strain appears to have replaced the previously predominant strain, GII.4 New Orleans. Compared with other norovirus genotypes, GII.4 noroviruses have been associated with increased rates of hospitalizations and deaths during outbreaks (5). Health-care providers and public health practitioners should remain vigilant to the potential for increased norovirus activity in the ongoing season related to the emergent GII.4 Sydney strain. Continued surveillance for norovirus outbreaks through CaliciNet and additional data on clinical and epidemiologic features of outbreaks collected through the National Outbreak Reporting System (NORS)§ will enable further assessment of the public health implications of the new GII.4 Sydney strain, including any association with increased severity or level of activity in the ongoing 2012–13 winter norovirus season. Proper hand hygiene, environmental disinfection, and isolation of ill persons remain the mainstays of norovirus prevention and control (1).

## Footnotes

* Additional information available at http://www.hpa.org.uk/NewsCentre/NationalPressReleases/2013PressReleases/130109Norovirusnewstrain.

† Data for 2012 are incomplete.

§ Additional information available at http://www.cdc.gov/nors.
